# Performance of Xpert MTB/RIF Ultra for diagnosis of pulmonary and extra-pulmonary tuberculosis, one year of use in a multi-centric hospital laboratory in Brussels, Belgium

**DOI:** 10.1371/journal.pone.0249734

**Published:** 2021-04-08

**Authors:** Leila Mekkaoui, Marie Hallin, Françoise Mouchet, Marie-Christine Payen, Evelyne Maillart, Philippe Clevenbergh, Aspasia Georgala, Sigi Van den Wijngaert

**Affiliations:** 1 Department of Microbiology, Laboratoire Hospitalier Universitaire de Bruxelles–Universitair Laboratorium Brussel (LHUB-ULB), Brussels, Belgium; 2 Pediatric Department, CHU Saint Pierre, Université Libre de Bruxelles, Brussels, Belgium; 3 Department of Infectious Diseases, CHU Saint-Pierre, Université Libre de Bruxelles, Brussels, Belgium; 4 Department of Infectious Diseases, Hôpital Universitaire Brugmann, Université Libre de Bruxelles, Brussels, Belgium; 5 Department of Infectious Diseases, Institut Jules Bordet, Université Libre de Bruxelles, Brussels, Belgium; The University of Georgia, UNITED STATES

## Abstract

Among the challenges in controlling tuberculosis, a rapid and accurate diagnostic test for the detection of *Mycobacterium tuberculosis* complex (*MTB*c) and its resistance to first line therapies is crucial. We evaluated the performance of the Xpert MTB/RIF Ultra assay (Xpert Ultra) for the rapid detection of *MTB*c and rifampicin resistance (RR) in 1120 pulmonary and 461 extra-pulmonary clinical specimens and compared it with conventional phenotypic techniques. The Xpert Ultra assay detected *MTB*c in 223 (14.1%) samples with an overall sensitivity and specificity, using culture as the “gold standard”, of 91.1% (95% CI, 85.6–95.1) and 94.5% (95% CI, 93.1–95.6), respectively. The sensitivity of the Xpert Ultra test for smear-negative extra-pulmonary specimens was high (87.1%), even higher than with smear-negative pulmonary specimens (81.8%). But this enhanced sensitivity came with a low overall specificity of smear-negative extra-pulmonary specimens (66.7%). For 73 patients, 79/1423 (3.4%) negative mycobacterial culture samples were found to be positive with Xpert Ultra. Clinical data was necessary to correctly interpret potential false-positive results, especially trace-positive results. Sensitivity of the Xpert Ultra to detect RR compared to drug susceptibility testing was 100% (95% CI, 29.2–100) and specificity was 99.2% (95% CI, 95.8–100). We concluded that the Xpert Ultra test is able to provide a reliable TB diagnosis within a significantly shorter turnaround time than culture. This is especially true for paucibacillary samples such as smear-negative pulmonary specimens and extra-pulmonary specimens.

## Introduction

Tuberculosis (TB), caused by *Mycobacterium tuberculosis* complex (*MTB*c), remains a global public health challenge with an estimated 10 million new cases and 1.4 million deaths in 2019 [1]. As in other countries with a low incidence of TB, the distribution of cases in Belgium is uneven and there are more cases in areas where vulnerable populations are concentrated. Thus, while the incidence of TB in Belgium in 2018 was of 8.6 per 100,000 inhabitants, the incidence of TB in Brussels was of 29.5 per 100,000 inhabitants and the proportion of multidrug-resistant (MDR) TB cases among new cases was of 2.55% [2].

For public health purposes, TB disease has been classified into pulmonary TB (PTB) which involves the lung parenchyma and is contagious and extra-pulmonary TB (EPTB) which involves organs other than lungs and is reported to account for 16% of all TB cases [1]. It has a poorer outcome in human immunodeficiency virus (HIV) co-infected patients.

Diagnosis of TB is based on clinical, radiological, histological and bacteriological findings (smear examination, molecular biology and culture). However, culture remains the reference standard for laboratory confirmation of TB disease. In addition, bacterial growth is required for drug susceptibility testing (DST). Unfortunately, results generally take several weeks due to slow growth pattern. Late diagnosis and delays in susceptibility testing lead to increased morbidity, mortality and ongoing transmission of the disease. Among the challenges in controlling TB, a rapid and accurate diagnostic test for the detection of *MTB*c and its resistance to first line therapies is crucial.

Therefore, the World Health Organization (WHO) has endorsed the implementation of rapid molecular laboratory methods for the diagnosis of PTB, such as the GeneXpert MTB/RIF (Xpert) (Cepheid, Sunnyvale, CA, USA) assay in December 2010 [3, 4]. The Xpert is an automated nucleic acid amplification test (NAAT), which requires minimal biosafety facilities and training for a simultaneous detection of genetic material of *MTB*c and mutations associated with rifampicin resistance (RR), an indicator of MDR-TB, in less than 2 hours. Since 2017, an improved version of the Xpert called Xpert MTB/RIF Ultra (Xpert Ultra), detecting *MTB*c in sputum with detection limit of 15.6 CFU/mL, has been implemented [5, 6]. Several studies have demonstrated that Xpert Ultra has a higher sensitivity but a lower specificity than Xpert in pulmonary samples, especially in paucibacillary disease, and provides a more-reliable detection of RR [6–8]. However, there are insufficient data on the performance of the Xpert Ultra in extra-pulmonary samples.

The aim of this study is to evaluate the performance of the Xpert MTB/RIF Ultra assay for the rapid detection of *MTB*c and RR in pulmonary as well as extra-pulmonary clinical specimens in our setting and to compare it with conventional phenotypic techniques.

## Materials and methods

### Patient population and data collection

We analyzed retrospectively data from patients suspected to have active PTB or EPTB attending one of the tertiary care hospitals (Centre Hospitalier Universitaire Saint-Pierre, Hôpital Universitaire Brugmann, Hôpital Universitaire des Enfants Reine Fabiola and Institut Jules Bordet) in Brussels, Belgium between 1 July 2018 and 30 July 2019 for whom at least one clinical sample was sent to the microbiology department for the detection of *MTB*c by conventional bacteriological methods (microscopy and culture) and by Xpert Ultra.

The data collected consisted of (i) sociodemographic characteristics (gender, age, ethnic or national origin, address), (ii) clinical information obtained from medical records (previously diagnosed TB and antituberculosis therapy [ATT], tuberculin skin test [TST], history of TB contact, HIV status, immunosuppressive treatment, antibiotic therapy), (iii) clinical evidence of TB (cough for more than 2 weeks, weight loss, persistent low-grade fever, night sweats, and/or lymphadenopathy), (iv) radiological evidence of TB, (v) all microbiological and other laboratory results (results of mycobacterial culture, acid-fast bacilli [AFB] smear, Xpert Ultra, interferon-_Ɣ_ release assay [IGRA], and histological results), and (vi) TB diagnostic confirmation (disease location) determined by Infectious Diseases or Pulmonology specialists.

Sensitivity, specificity, positive predictive value (PPV), and negative predictive value (NPV) of the Xpert Ultra were calculated, using culture as the “gold standard” for the diagnosis of confirmed TB. Discrepant results were analyzed based on patient’s data collected and the final diagnosis established by the physicians in charge.

### Samples processing and laboratory methods of *MTB*c detection

The pulmonary samples (sputum, bronchotracheal aspirate, bronchoalveolar lavage [BAL], pulmonary biopsies and gastric fluid specimens) and extra-pulmonary samples (cerebrospinal fluid [CSF], lymph node samples, tissue biopsies, skin biopsies, osteoarticular samples, pus, pleural punctures or biopsies, urinary samples and other localizations) obtained from patients suspected of having TB were included in this study and handled in the Biosafety Level 3 laboratory of the “Laboratoire Hospitalier Universitaire de Bruxelles—Universitair Laboratorium van Brussel” (LHUB-ULB). Nonsterile clinical specimens (all samples except bone marrow and CSF) were processed by the conventional *N*-acetyl-*L*-cysteine-sodium hydroxide (NALC-NaOH) digestion and decontamination method. Briefly, samples were transferred to a falcon tube and were decontaminated by the addition of an equal volume of NALC-NaOH. After 30 minutes, phosphate buffer was added to a final volume of 50 mL to minimize the continuing action of NaOH and to lower the pH of the specimen. Samples were concentrated by centrifugation for 15 minutes at 3,000 g. The falcon tubes were kept for 5 min to allow aerosols to settle down. The supernatant was decanted and the concentrated sediment was resuspended into 2–3 mL of phosphate buffer. The resulting NALC/NaOH pellet was tested by trained laboratory technicians using two methods: (i) auramine fluorescence smear microscopy, (ii) microbiological growth indicator tubes (MGIT) liquid culture and Lowenstein-Jensen (LJ) solid culture.

#### Auramine fluorescence smears microscopy

After decontamination, AFB smears were prepared according to the Fluorochrome Auramine O staining technique recommended by WHO (1998). In brief, smears were sprayed with auramine O for 5–10 min and destained with acid alcohol for 1 min. With auramine O staining, *Mycobacteria* appear as bright yellow fluorescent rods on a dark background. Quantification of AFB was reported using the Centers for Disease Control and Prevention scoring system.

#### Cultures

The liquid culture media was based on fluorometric detection of growth. MGIT tubes were supplemented with 1 mL of PANTA. Then 0.5 mL of decontaminated/sterile specimens was inoculated into the MGIT tube. The tubes were incubated in the automated liquid culture BACTEC MGIT 960 system (Becton Dickinson, Sparks, Diagnostic Systems, USA) semi-automated liquid culture system machine for a maximum incubation time of 8 weeks at 35–38°C. Solid culture media, Lowenstein-Jensen (LJ), was inoculated with 0.5 mL of decontaminated/sterile specimens and incubated at 37°C for a maximum incubation time of 10 weeks. Deficiency of growth at the end of the 8^th^ and 10^th^ week was regarded as a negative culture.

#### Identification and DST of *MTB*c strains

For all first positive cultures, species confirmation and DST were performed according to the manufacturer’s recommendations using respectively a rapid immuno-chromatographic device (SD TB Ag MPT 64 Rapid, Bioline, Republic of Korea); and the Bactec MGIT 960 SIRE and Bactec MGIT 960 pyrazinamide kits (Becton Dickinson-Sparks, Maryland, USA) including isoniazid (INH), rifampicin (RIF), ethambutol (EMB) and pyrazinamide (PZA) antituberculous drugs. Strains resistant to INH and RIF were considered as MDR. For all MPT 64-negative strains, identification was performed using the MALDI-TOF MS and were confirmed by the Belgian National Reference Center (NRC) for tuberculosis and mycobacteria (https://www.sciensano.be/en/projects/national-reference-center-tuberculosis-and-mycobacteria).

#### GeneXpert MTB/RIF Ultra assay

The Xpert Ultra assay was performed on samples as recommended by the manufacturer. But following requests of additional analysis by clinicians and limited quantity of sample available, the Xpert Ultra assay was performed on the NALC/NaOH pellet. The samples were diluted with sample reagent using a ratio of 1:2. This mixture was manually agitated twice for at least 10 seconds and incubated for 15 minutes at room temperature. Two mL of the mixture was then transferred to the Xpert Ultra MTB/RIF disposable plastic cartridge. The MTB/RIF cartridge, which integrates nucleic acids extraction and PCR, is then inserted into the Xpert instrument. Xpert Ultra uses a hemi-nested PCR to amplify the rifampin resistance-determining region (RRDR) of the MTB*c rpoB* gene. The results are automatically reported by the instrument within 80 minutes, and for *MTB*c detection are divided into six categories including high, medium, low, very low, *MTB*c trace, and not detected. The “trace” category is designed to identify samples with the lowest bacillary burden detected, which are IS6110/IS1081 (multicopy sequences shown to have high sensitivity for diagnosing TB) positive but *rpoB* negative [6]. The Xpert Ultra reports RR result as detected or not detected for all categories of *MTB*c positive samples except the ‘trace’ category, for which the RR result is reported as indeterminate, as quantity of *MTB*c DNA is too low to conclude.

### Ethics committee approval

The study was approved by the Medical Ethics Committees of the four hospitals.

### Statistical analysis

Sensitivity, specificity and predictive values with 95% confidence intervals (95% CI) were calculated using *MTB*c culture as gold standard. Statistical analyses were performed using GraphPad Prism 6.00 Software, *Inc*, San Diego, USA. Descriptive statistics were used to characterize the study population; normally distributed continuous data were summarized by means ± standard deviation and non-normally distributed continuous data by medians and interquartile range (IQR). The analysis of categorical variables was examined by the Pearson chi-square test. A value of *P* of < 0.05 was considered significant for all statistical analysis.

## Results

### Study population

During the study period, a total of 1581 samples from 1270 patients suspected of having TB were analyzed. For some patients, 2 samples of different natures (1 PTB and 1 EPTB) were collected. The Xpert Ultra has never been performed on duplicate samples. Thirty seven samples were obtained from 31 children (<15 years old), 16 females and 15 males (median age: 4.02 ± 7.98 years), and 1544 samples were obtained from 1239 adults (≥15 years old), 400 females and 839 males (median age: 52.46 ± 28.49 years).

### Specimens

Pulmonary samples represented 1120 out of 1581 (521 sputa, 12 oropharyngeal aspirations, 65 bronchial aspirations, 483 BAL, 23 pulmonary biopsies and 16 gastric fluids specimens). The remaining 461 specimens were from extra-pulmonary origin (196 lymph nodes [22 fluids and 174 biopsies], 53 tissue biopsies [18 skin, 21 digestive and 14 others localizations biopsies], 77 pleural samples [65 fluids and 12 biopsies], 19 pericardial samples [14 fluids and 5 biopsies], 34 pus aspirations [24 ascitic, 2 ocular and 8 other localizations fluids], 51 CSF, 27 osteoarticular samples [19 bone biopsies and 8 synovial fluids], 4 urine samples).

### Performance of the Xpert MTB/RIF Ultra assay and microscopy to detect *MTB*c

**Fig 1:**
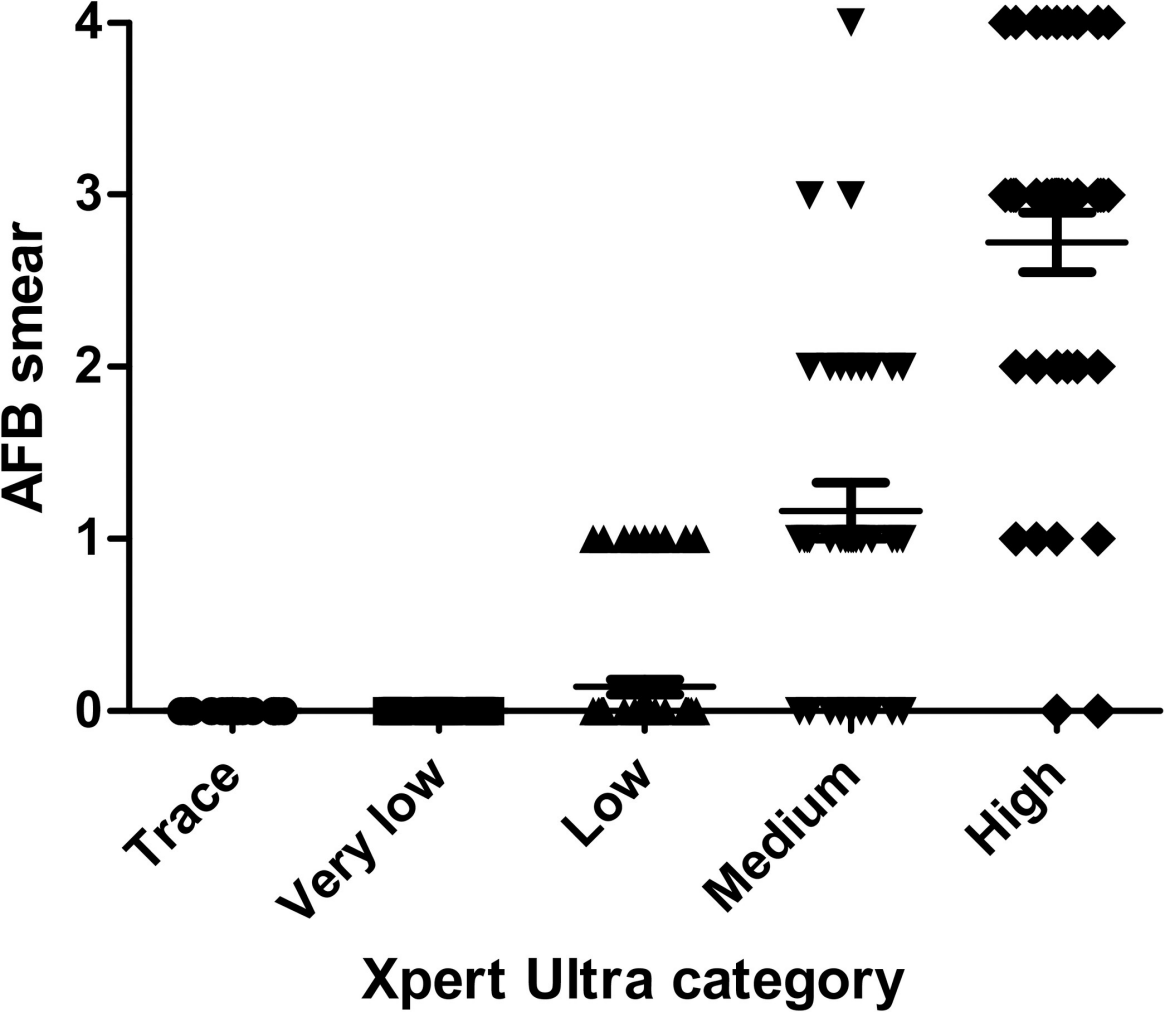
Correlation between Xpert Ultra categories and AFB smear microscopy score. *P* < 0,0001.

The GeneXpert MTB/RIF Ultra assay for the detection of *MTB*c was positive in 223 (14.1%) samples and negative in 1358 (85.9%) samples. Nine positive samples (4.04%) were obtained from children and 214 from adults (95.96%). The results by specimen type of culture, microscopy, Xpert Ultra and the diagnostic performance of Xpert Ultra with culture as the reference standard are summarized in Table 1. The Xpert Ultra was positive for 144 (trace [n = 10], very low [n = 20], low [n = 42], medium [n = 32], and high [n = 40]) of 158 culture-positive samples and 79 (trace [n = 36], very low [n = 17], low [n = 22], and medium [n = 4]) of 1423 culture-negative samples. All specimens with Xpert very low or trace positive were smear-negative (Fig 1). The Xpert Ultra was negative for 1344 of 1423 culture-negative samples and for 14 of 158 culture-positive samples. The false-negative Xpert Ultra results (10 pulmonary and 4 extra-pulmonary samples) were obtained from 13 adults and 1 child.

**Table 1:** Results by specimen type of *MTB*c culture, smear microscopy, Xpert Ultra and diagnostic performance of Xpert Ultra with culture as the reference standard.

Type of specimen	n	Culture (+) (n = 158)				Culture (-) (n = 1423)				Performance of Xpert Ultra			
		smear (+)		smear (-)		smear (+)		smear (-)		Sensitivity (%) (95% CI)	Specificity (%) (95% CI)	PPV (%) (95% CI)	NPV (%) (95% CI)
		XU (+) (n = 72)	XU (-) (n = 0)	XU (+) (n = 72)	XU (-) (n = 14)	XU (+) (n = 2)	XU (-) (n = 10)	XU (+) (n = 77)	XU (-) (n = 1334)				
Sputum	521	58	0	35	7	1	5	12	403	93 (86,1–97,1)	96,9 (94,8–98,4)	87,7 (79,9–93,3)	98,3 (96,6–99,3)
BAL and RA	560	5	0	10	2	0	3	29	511	88,2 (63,6–98,5)	94,7 (92,4–96,4)	34,1 (20,5–49,9)	99,6 (98,6–100)
Gastric fluid	16	0	0	0	1	0	0	3	12	NA	80 (51,9–95,7)	NA	92,3 (64–99,8)
Pulmonary biopsy	23	0	0	0	0	0	0	0	23	NA	NA	NA	NA
Lymph node	196	7	0	16	1	0	1	24	147	95,8 (78,9–99,9)	86,1 (80–90,9)	48,9 (34,1–63,9)	99,3 (96,3–100)
Tissue biopsy	53	0	0	0	0	0	1	2	50	NA	NA	NA	NA
Pleural fluid	77	0	0	6	3	0	0	1	67	66,7 (29,9–92,5)	98,5 (92,1–100)	85,7 (42,1–99,6)	95,7 (88–99,1)
Pericardial fluid	19	0	0	0	0	0	0	0	19	NA	NA	NA	NA
CSF	51	0	0	0	0	0	0	2	49	NA	NA	NA	NA
Osteoarticular	27	2	0	1	0	1	0	2	21	100 (29,2–100)	87,5 (67,6–97,3)	50 (11,8–88,2)	100 (83,9–100)
Pus	34	0	0	4	0	0	0	2	28	100 (39,8–100)	93,3 (77,9–99,2)	66,7 (22,3–95,7)	100 (87,7–100)
Urine	4	0	0	0	0	0	0	0	4	NA	NA	NA	NA

XU, GeneXpert MTB/RIF Ultra assay; PPV, positive predictive value; NPV, negative predictive value; CI, confidence interval; BAL and RA, bronchoalveolar lavage and respiratory aspirate; NA, not applicable; CSF, cerebrospinal fluid.

The overall diagnostic sensitivity of Xpert Ultra to detect *MTB*c was 91.1% (95% CI, 85.6–95.1), specificity was 94.5% (95% CI, 93.1–95.6), NPV was 99% (95% CI, 98.3–99.4), and PPV was 64.6% (95% CI, 57.9–70.8). The sensitivity, specificity, NPV, and PPV of the test for smear-positive and smear-negative pulmonary and extra-pulmonary samples are detailed in Table 2. The sensitivity, specificity, NPV, and PPV of Xpert Ultra to detect *MTB*c in pediatric population were 50% (95% CI, 12.58–98.74), 77.14% (95% CI, 59.86–89.58), 96.43% (95% CI, 81.65–99.91), and 11.11% (95% CI, 0.28–48.25) respectively.

**Table 2:** Diagnostic performance of Xpert Ultra and smear using culture as the reference standard.

Method	Pulmonary samples (n = 1120)				Extra-pulmonary samples (n = 461)			
	Sensitivity (%)	Specificity (%)	PPV (%)	NPV (%)	Sensitivity (%)	Specificity (%)	PPV (%)	NPV (%)
	(95% CI)	(95% CI)	(95% CI)	(95% CI)	(95% CI)	(95% CI)	(95% CI)	(95% CI)
Smear	53,4	99,1	87,5	94,8	22,5	99,3	75	93,1
	(44–62,6)	(98,3–99,6)	(77,6–94,1)	(93,2–96)	(10,8–38,5)	(97,9–99,9)	(42,8–94,5)	(90,3–95,3)
Xpert Ultra assay (all samples)	91,5	95,5	70,6	99	90	91,9	51,4	99
	(85–95,9)	(94–96,7)	(62,7–77,7)	(98,1–99,5)	(76,3–97,2)	(88,9–94,3)	(39,1–63,6)	(97,4–99,7)
Smear positive	100	88,9	98,4	100	100	66,7	90	100
	(94,3–100)	(51,8–99,7)	(91,6–100)	(63,1–100)	(66,4–100)	(9,4–99,2)	(55,5–99,8)	(15,8–100)
Smear negative	81,8	95,6	50,6	99	87,1	92,1	45	99
	(69,1–90,9)	(94,1–96,8)	(39,8–61,3)	(98,1–99,5)	(70,2–96,4)	(89,1–94,5)	(32,1–58,4)	(97,4–99,7)

PPV, positive predictive value; NPV, negative predictive value; CI, confidence interval.

Additionally, non-tuberculous mycobacteria (NTM) were recovered from 28 (24 pulmonary and 4 extra-pulmonary) samples, of whom 6 (20.7%) were smear-positive, *MTB*c culture- and Xpert Ultra-negative; 21 were smear-, *MTB*c culture- and Xpert Ultra-negative; 1 was smear-negative, *MTB*c and NTM culture-positive, and Xpert Ultra-negative. The mycobacteria isolated included *M*. *gordonae* (n = 4), *M*. *xenopi* (n = 9), *M*. *avium* (n = 4), *M*. *chelonae abscessus* (n = 1), *M*. *chimaera-intracellulare group* (n = 5), *M*. *kansasii* (n = 5).

### Analysis of false positive results compared to patient’s data collected

The 79 false-positive Xpert Ultra results were obtained from 73 patients (7/73 were children and 10/73 were HIV-seropositive). The large majority (>90%) presented a clear clinical evidence of TB (e.g. cough for more than 2 weeks, weight loss, night sweat, persistent low-grade fever, lymphadenopathy…) and a medical imaging suspect of active TB. Of note, 27 of them (37%) had a *MTB*c identified on a follow-up culture and/or Xpert Ultra assay. Furthermore, 20 of the 73 patients had a history of TB and had been treated. Of those, 12 presented with active or reactivated TB due to incomplete or incorrect ATT. The final diagnosis established by the clinician were: 60/73 active TB including 18 ganglionic, 9 disseminated, 3 abdominal, 2 pleural, 2 meningeal, and 1 osteoarticular TB, 12/73 non-active TB (8/12 were previously treated TB cases), and 1/73 patient was lost to follow-up. All TB cases were treated with ATT. The clinical and demographic characteristics of patients with a false-positive Xpert Ultra result and a final diagnosis of active TB are summarized in Table 3.

**Table 3:** Clinical and demographic characteristics of patients with apparent false-positive Xpert Ultra results and a final diagnosis of active TB.

Characteristic	With other *MTBc*-positive result^a^		Without any other *MTBc*-positive result^a^	
	PTB cases	EPTB cases	PTB cases	EPTB cases
	(n = 12)	(n = 15)	(n = 13)	(n = 20)
Gender				
Male	12	6	11	12
Female	0	9	2	8
Age group (years)				
Children (<15)	1	0	1	3
Adults (≥15)	11	15	12	17
Origin				
Born in Belgium	3	1	3	1
Non-Belgium-Born	9	14	10	19
Previous TB history	2	4	2	4
TB contact history	8	3	6	8
Immunosuppressive treatment	1	0	0	1
Other testing performed ^b^ ^and^ ^c^				
Histological pattern of TB	0	5	0	6
TST +	1	2	1	2
IGRA +	0	1	0	0
HIV +	0	4	2	2

PTB, pulmonary tuberculosis; EPTB, extra-pulmonary tuberculosis; TST, tuberculin skin test; IGRA, interferon-Ɣ release assay; HIV +, HIV-seropositive.

^a^*MTBc* result including follow-up culture and/or Xpert Ultra result.

^b^One patient had both TST and IGRA +.

^c^One patient had both histological pattern of TB and TST +.

### Performance of the Xpert MTB/RIF Ultra assay to detect RR-*MTB*c

During the study 144 *MTB*c DST were performed. Five PZA mono-resistant-strains were identified as 1 *M*. *africanum*, 1 *M*. *tuberculosis* and 3 *M*. *bovis ssp bovis*. For one sample, the DST was indeterminate due to a mix of a *MTB*c and a *M*. *chimaera-intracellulare* group strain in the culture.

RR assay results of Xpert Ultra compared to DST are shown in Table 4. Of the 223 samples with a *MTB*c positive Xpert Ultra test, 5 (2.24%) showed “RR detected”, 172 (77.13%) “RR not detected” and the 46 samples with *MTB*c “traces” (20.63%) were replied as “RR indeterminate”. RR was only observed in pulmonary samples (3 sputa and 2 BAL) of adult males. Four patients were from Central and Eastern Europe and one from Ethiopia. The Xpert Ultra positive results were in accordance with the DST results for four patients. The DST performed on the isolate from the last sample, reported as RR by Xpert Ultra showed to be RIF-susceptible, but INH+EMB+PZA-resistant. It was sent to the NRC which confirmed the presence of a LEU533Pro mutation, conferring a low-level resistance to RIF. One RR isolate was missed by the Xpert Ultra test, as the *MTB*c result of the sample was “traces”, RR detection result was “indeterminate”.

**Table 4:** RR assay results of Xpert Ultra compared to DST.

DST	Xpert Ultra RR detection			Total
	Not detected	Detected	Indeterminate	
Multi S	117	0	9	126
RR	0	1	0	1
RIF+INH R	0	2	0	2
INH R	4	0	1	5
INH+EMB R	3	0	0	3
INH+EMB+PZA R	0	1^a^	0	1
PZA R	5	0	0	5
Indeterminate	1	0	0	1
Total	130	4	10	144

Multi S, multi-sensitive strain; RR, rifampicin resistance; DST, drug susceptibility testing; RIF, rifampicin; INH, isoniazid; EMB, ethambutol; PZA, pyrazinamide.

^a^ Presence of a LEU533Pro mutation conferring a low-level resistance to RIF.

Sensitivity of the Xpert Ultra to detect RR compared to MGIT DST was thus 100% (95% CI, 29.2–100), specificity was 99.2% (95% CI, 95.8–100), NPV was 100% (95% CI, 97.2–100), and PPV was 75% (95% CI, 19.4–99.4).

### Time to positivity of culture according to Xpert Ultra and microscopy results

The laboratory processing time varied between 2 to 24h for the Xpert Ultra and 8 to 24h for smear microscopy. The mycobacteriology department is only active during office hours.

**Fig 2:**
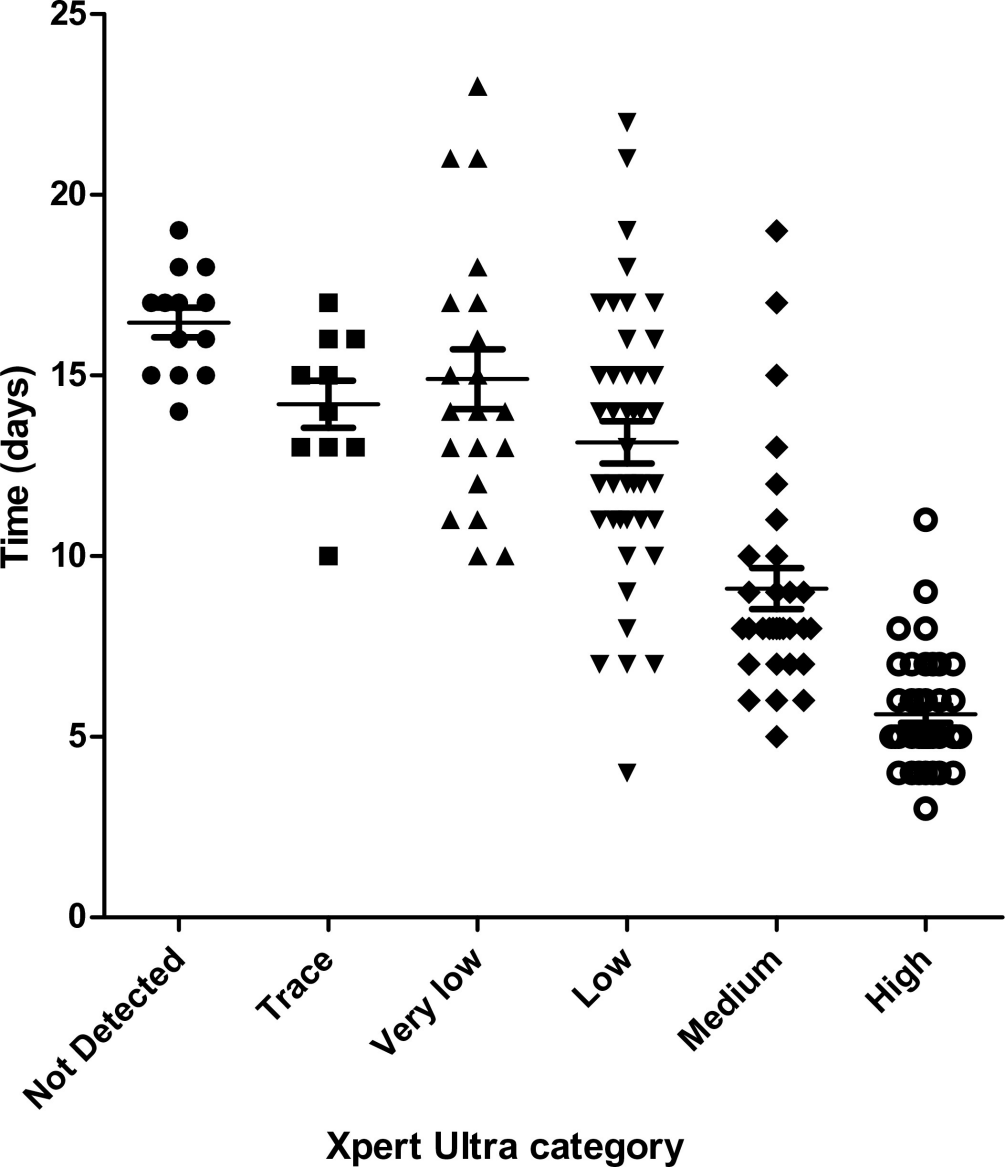
Correlation between Xpert Ultra categories and time to positivity of *MTBc* culture. *P* < 0,0001.

The mean time-to-positivity of culture among Xpert Ultra true-positive samples was 10.48 ± 4.68 days. The mean time-to-positivity of culture among Xpert Ultra true-positive and smear-negative samples was 13.91 ± 3.72 days (13.78 ± 3.95 days for pulmonary and 14.15 ± 3.36 days for extra-pulmonary samples), and for Xpert Ultra true-positive and smear-positive samples was 6.56 ± 1.97 days for pulmonary and 10 ± 3.39 days for extra-pulmonary samples. By contrast, the mean time-to-positivity among the Xpert Ultra false-negative samples was 16.71 ± 1.68 days (16.67 ± 1.32 days for pulmonary and 16.8 ± 2.39 days for extra-pulmonary samples). Fig 2 shows these times to positivity of cultures according to the Xpert Ultra results.

## Discussion

In the present study, we evaluated the analytical performance of Xpert MTB/RIF Ultra assay on more than 1500 pulmonary and extra-pulmonary specimens, collected in the cosmopolite urban setting of Brussels. Even if this technique is not currently approved or available for use in many countries, we have implemented it in July 2018.

This study has several limitations. First, although sample diversity was high, the limited number of specific sample types (e.g. CSF, osteoarticular, gastric fluids or pleural samples) did not allow for proper assessment of the Xpert Ultra performance for these sample categories. A second limitation was the availability of certain clinical data, such as previous history of TB or HIV status, which was limited due to the retrospective nature of our data collection. In Belgium, the data regarding previous history of TB is known to be missing in 15.9% of the TB cases (mainly Non-Belgium-Born patients) [2]. A third limitation was that the Xpert Ultra assay was performed on the NALC/NaOH pellet for 53/1581 samples (3.4%) in this study. This could introduce a possible bias due to the effect of dilution, but on the other hand, there is a concentration step in this procedure. This method has been evaluated before the implementation of Xpert Ultra in our laboratory, with no impact on sensitivity of semi-quantitative results. A final limitation was that usually a minimum of 2 mL of CSF is required for Xpert Ultra testing, but in clinical practice, other routine analyses must be performed on the same CSF sample and thus 2 mL are not always available to process Xpert Ultra. These variations in the volumes of CSF tested could have an impact on the test performance.

The NPV value obtained in this study was very high (99%), in both pulmonary and extra-pulmonary specimens. According to the WHO, this NPV indicates the ability of Xpert Ultra to formally exclude a diagnosis of TB. Nevertheless, 14 cases were missed by Xpert Ultra and by microscopy screening. Those results showed that a negative Xpert Ultra result cannot completely rule out an active TB, especially in cases of suspected pleural TB.

Previous studies have reported test sensitivities of 63 to 91.7% for smear-negative, culture-positive PTB cases, and 98.9 to 100% for smear-positive, culture-positive PTB cases, while the test specificity were at 95.6 to 98.7% [6–9]. In line with those studies, the sensitivity and the specificity of the Xpert Ultra retrieved in the present study were 81.8% and 95.6% for smear-negative and 100% and 88.9% for smear-positive pulmonary specimens, respectively. The fewer studies reporting the specific performance of the Xpert Ultra among pediatric PTB showed sensitivities of 64.3 to 75.3% and specificities of 96.9 to 98.1% [10, 11]. In the present study, the overall sensitivity and specificity among our pediatric population were 50% and 77.1% respectively. However, the limited number of children in this study did not allow to assess properly the Xpert Ultra performance for pediatric samples.

The sensitivity of the Xpert Ultra test for smear-negative extra-pulmonary specimens was high (87.1%), even higher than with smear-negative pulmonary specimens. This is of particular clinical importance as a large bacillary load (10^3^/mL) is required for a smear to become AFB positive [12]. As described in the study of Zeka et al. [13], this could be the result of a low number of organisms in extra-pulmonary samples. In the present study, for example, smear had a low sensitivity for pulmonary specimens (53.4%) and even lower for extra-pulmonary specimens (22.5%). But this enhanced sensitivity came with a low overall specificity of smear-negative extra-pulmonary specimens (66.7%) compared with culture. We did not perform a repeat testing on the same specimen for “trace” results, which could reduce some loss of specificity, as supported by Kendall et al. and Piersimoni et al. [14, 15].

Considering those factors, the Xpert Ultra assay is a useful confirmatory diagnostic test for EPTB from various types of clinical specimens, such as CSF and tissue samples. Of note, the sensitivity and the specificity varied greatly between different sample types, as illustrated by the 14 culture-positive samples that had a negative Xpert Ultra result: 10 pulmonary (8% of the positive pulmonary specimens), 1 lymph node (4%) and 3 pleural specimens (33% of the positive specimens). Pleural TB is the most common cause of pleural effusion in many developing countries and remains a diagnostic challenge due to unreliable laboratory detection test results [16]. Numerous studies [17–20] reported poor sensitivity (29 to 55%) for testing of pleural effusion specimens. This could be due to very low bacillary load, the presence of inhibitory substance or the use of different gold standards in various studies. Our results are not in line with those of the Saeed et al. study [21], conducted in Pakistan, in which inclusion of patients with severe forms of pleural TB could have contributed to high sensitivity in pleural fluids (90%). The sensitivity of the Xpert Ultra assay in pleural specimens should be accurately assessed in a larger population.

For 73 patients, 79/1423 (3.4%) negative mycobacterial culture samples were found to be positive with Xpert Ultra. But only 12 (16%) of these patients were “clinically” diagnosed with non-active TB, of which 8 had a history of treated TB. The detection of DNA from dead bacilli can easily explain the fact that Xpert Ultra remains positive (trace [n = 7] and very low [n = 1]) for months despite adequate treatment [22, 23]. The 4 remaining patients had no known history of TB and a positive Xpert Ultra (trace [n = 4] in BAL samples). These results may be due to environmental contamination of the samples. However, Arend et al. [24] assumed that TB should be viewed as a spectrum of diseases with subclinical, self-contained TB or even a latent TB. This is another possible source of a supposedly false-positive *MTB*c DNA detection in sputum.

Thus, even though the mycobacterial culture was the reference technique, the Xpert Ultra contributed to the diagnosis of active TB (mainly ganglionic, meningeal, abdominal and osteoarticular TB cases) for the large majority (82.2%) of our patients with clear clinical evidence of disease but negative culture. The negativity of the culture in these cases could be related to (i) the taking of antibiotics before sampling, as in osteoarticular or abdominal TB, for which TB is most often considered as a last resort diagnostic, after antibiotic therapy failure, (ii) the amount of tubercle bacilli (among the 60 active culture-negative TB cases, there were 25 “trace” and 16 “very low” Xpert Ultra results), (iii) the nature of the samples (culture is known to be imperfect for meningitis and osteoarticular TB, and detection of *MTB* DNA should always be considered as significant) [25–28], and (iv) the quality of the samples (sampling methods used, dilution, transport conditions and delays, over-decontamination, and environmental contamination). Otherwise, the mycobacterial culture should not be the only reference standard for the diagnosis of tuberculous meningitis. As shown in the study of Donovan et al. [29], the choice of clinical or mycobacterial culture reference standards affect the sensitivity of the test (47.2% or 90.9% respectively). Moreover, our results and those described in the study of Chin et al. [30] are not in line with those of the Donovan et al. study in which Xpert Ultra did not have a higher rate of detection of *MTB*c in CSF by compared to smear microscopy and culture. This discrepancy in the study of Donovan et al., might be attributed to the use of the sediment after centrifugation of a large-volume CSF to perform Xpert Ultra.

A positive correlation between the Xpert Ultra semi-quantitative results and AFB burden was found, as well as time-to-positivity of the culture; similarly, longer TAT for Xpert Ultra-negative samples could be due to the low bacillary load which were under the limits of detection of the test. As described by Opota et al. [7], patients with Xpert Utra “very low” and “trace” results correspond to smear-negative patients, which correspond to a limited transmission potential. In this regard, recent studies have been performed to evaluate new strategies which use one single Xpert to guide discontinuation of respiratory isolation for hospitalized patients with presumptive TB [31, 32]. Even if the Xpert Ultra demonstrated superiority to smear in microbiologically confirmed TB, the addition of smear microscopy would be beneficial to detect NTM, which may have clinical relevance. In our study, smear-positive but Xpert Ultra-negative samples were assumed to be positive for NTM weeks before the culture results were available.

A limited number of RR were collected in the present study (2.44%), which is in accordance with the reported national register of 2018 (FARES) [2]. A major advantage of the Xpert Ultra is the ability to rapidly identify RR which is very useful for patients infected with RIF- and INH-resistant, or RIF-resistant and INH-susceptible TB because they should be treated with second-line therapy. As described in previous studies, the Xpert Ultra had a high performance for detection of RR, with a 100% sensitivity and 99.2% specificity. In a mixed infection with both a *MTB*c and a *M*. *chimaera-intracellulare group*, the phenotypic DST result was indeterminate and Xpert Ultra showed the presence of RIF-susceptible TB. This finding was also described by Chakravorty et al. [6], indicating that high background levels of NTM do not significantly interfere with the performance of the Xpert Ultra. Moreover, for one RR-strain by Xpert Ultra, a RIF susceptible phenotypic DST was obtained and further analysis showed a LEU533Pro mutation by sequencing, which confers a low-level resistance to RIF. Similar findings were observed in case of silent mutations in *rpoB* by Mathys et al. [33]. This study counted a lot of strains with a trace-positive Xpert Ultra results (n = 46), hence the RR could not be determined. Finally, the Xpert Ultra had obviously not the capacity to detect the other resistances: for 13 RIF-susceptible patients, 8 INH-resistant and 5 PZA-resistant TB were found, for whom efficacy of first-line therapy was sub-optimal and an appropriate treatment was not given from the start. Thus, despite its slowness, the phenotypic DST remains the gold standard for detection of drug-resistant *MTB*c.

## Conclusion

Diagnosis of TB disease remains challenging for health services and clinicians due to delayed diagnosis and management. In our clinical setting, the Xpert Ultra test has been able to provide a reliable TB diagnosis within a significantly shorter turnaround time than culture. This was especially true for paucibacillary samples such as smear-negative pulmonary specimens and extra-pulmonary specimens. However, clinical data was necessary to correctly interpret potential false-positive results, especially trace-positive results. Moreover, the limited information on the diagnostic validity of this test for several types of extra-pulmonary samples, underline the need for additional studies to confirm these observations.
